# Niche partitioning between close relatives suggests trade-offs between adaptation to local environments and competition

**DOI:** 10.1002/ece3.462

**Published:** 2013-01-24

**Authors:** Megan L Peterson, Kevin J Rice, Jason P Sexton

**Affiliations:** 1 Ecology and Evolutionary Biology, University of California Santa Cruz1156 High Street, Santa Cruz, CA, 95064; 2 Department of Plant Sciences and The Center for Population Biology, University of CaliforniaOne Shields Avenue, Davis, CA, 95616; 3 Department of Zoology, Bio21 Molecular Science Institute, The University of MelbourneParkville, Victoria, 3010, Australia

**Keywords:** Competition, facilitation, habitat partitioning, *Mimulus*, niche conservatism, niche evolution, spatial storage effect, species coexistence, species range limits, Stress Gradient Hypothesis

## Abstract

Niche partitioning among close relatives may reflect trade-offs underlying species divergence and coexistence (e.g., between stress tolerance and competitive ability). We quantified the effects of habitat and congeneric species interactions on fitness for two closely related herbaceous plant species, *Mimulus guttatus* and *Mimulus laciniatus*, in three common habitat types within their sympatric range. Drought stress strongly reduced survival of *M. guttatus* in fast-drying seeps occupied by *M. laciniatus*, suggesting that divergent habitat adaptation maintains this niche boundary. However, neither seedling performance nor congeneric competition explained the absence of *M. laciniatus* from shady streams where *M. guttatus* thrives. *M. laciniatus* may be excluded from this habitat by competition with other species in the community or mature *M. guttatus*. Species performance and competitive ability were similar in sympatric meadows where plant community stature and the growing season length are intermediate between seeps and streams. Stochastic effects (e.g., dispersal among habitats or temporal variation) may contribute to coexistence in this habitat. Habitat adaptation, species interactions, and stochastic mechanisms influence sympatric distributions for these recently diverged species.

## Introduction

Niche divergence among closely related species reflects both the selective forces underlying species divergence and the ecological mechanisms structuring current species distributions. Niche partitioning is driven by trade-offs in resource use that prevent the evolution of a single universal niche; such trade-offs are integral components of theory regarding distribution limits (e.g., Kirkpatrick and Barton 1997), ecological speciation (e.g., Rundle and Nosil 2005), and species coexistence (e.g., Tilman 2004). Trade-offs between adaptations to species interactions and other environmental conditions may be particularly important across environmental or resource gradients, where the relative importance of physiological tolerance and competitive ability may vary (Grime 1977; Gaucherand et al. 2006; Liancourt and Tielbörger 2009). Despite long-standing interest in the divergence, distribution, and coexistence of closely related species, a recent review (Sexton et al. 2009) notes an absence of experimental field tests of the relative strengths of species interactions and adaptation to other environmental conditions in setting niche boundaries between recently diverged species. Quantifying the effects of habitat and congeneric interactions in locally sympatric and allopatric habitats allows tests for trade-offs in their relative importance for the divergence and coexistence of closely related species.

Traditionally, species boundaries are generally thought to be maintained by species interactions in productive habitats and physiological limits in stressful habitats (e.g., Connell 1961; Gross and Price 2000). The realized niche is thus conceived as a sub-space of the fundamental niche (Hutchinson 1957); when abiotic mechanisms fail to explain persistent niche boundaries, biological mechanisms such as competition are invoked (e.g., Wethey 1983; Gross and Price 2000; Ettinger et al. 2011). Yet in stressful habitats, facilitative interactions may expand the realized niche to encompass a greater range of environmental conditions (Callaway 1995). Under the Stress Gradient Hypothesis (SGH), species interactions are predicted to switch from primarily facilitative in stressful habitats to primarily competitive in productive habitats (Bertness and Callaway 1994; Maestre et al. 2009). Support for the SGH has been found within communities (e.g., Choler et al. 2001; Liancourt et al. 2005), yet its importance for explaining evolutionary distribution limits (i.e., between closely related species) across environments has to our knowledge not yet been tested.

Niche divergence between closely related taxa across environmental gradients may reflect trade-offs between competitive ability and stress tolerance (Grime 1977; Liancourt et al. 2005; Liancourt and Tielbörger 2009; but see Emery et al. 2001). For example, rapid development in annual plant species may allow stress avoidance, but reduce competitive ability, whereas perennial species exhibit greater vegetative growth that may confer increased competitive ability, but decrease stress tolerance or avoidance (e.g., Tercek and Whitbeck 2004; Cui et al. 2011). Consequently, the strength and sign of species interactions may depend on both the environment in which they are measured and specific functional traits of the interacting taxa (Goldberg 1996).

Even if closely related species exhibit divergence across certain niche axes, other axes may be conserved (Holt 2009). Niche conservatism limits the coexistence of closely related species (MacArthur and Levins 1967). Alternatively, speciation may be associated with niche divergence (e.g., Evans Margaret et al. 2009), potentially reducing competition between close relatives in particular environments. The relative importance of competition with close or distant relatives in determining species distributions remains practically untested (but see Burns and Strauss 2011), and may be habitat-dependent.

When habitat partitioning occurs within the scale of dispersal, species coexistence in locally sympatric habitats may be maintained by either fluctuation-dependent or fluctuation-independent mechanisms (Chesson 2000). Fluctuation-independent mechanisms require that each species exhibits stronger intraspecific competition than interspecific competition independently of environmental variation (e.g., Tilman 1982). Alternatively, fluctuation-dependent mechanisms such as the storage effect rely on covariance between environmental conditions and the strength of competition, allowing intraspecific interactions to be spatially or temporally concentrated relative to interspecific interactions (Chesson and Warner 1981). For example, spatial variation allows positive population growth in favorable habitats to both concentrate intraspecific competition and buffer the effects of unfavorable habitats (Sears and Chesson 2007). In this case, dispersal from competitive refuges into nearby sympatric habitats may maintain species coexistence by preventing competitive exclusion (Amarasekare 2003).

**Figure 1:**
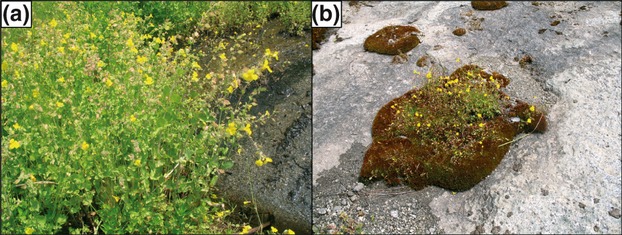
Closely related species of *Mimulus* exhibit fine-scale habitat partitioning. A: *Mimulus guttatus* in streams. B: *Mimulus laciniatus* in moss patches on granite seeps.

Here, we use two recently diverged plant species that exhibit persistent habitat partitioning to examine the mechanisms and trade-offs underlying niche divergence and local patterns of allopatry and sympatry. *Mimulus guttatus* thrives in perennial streams, whereas its close relative *Mimulus laciniatus* occupies nearby fast-drying seeps; either species is absent from the above habitat occupied by its congener (Fig. 1). Both species co-occur in meadow habitats with intermediate water availability. Habitat partitioning in this system occurs within the scale of dispersal, providing an opportunity to examine the mechanisms underlying persistent niche boundaries. We quantify habitat-specific responses and inter- and intraspecific interactions to test whether divergent adaptations to each habitat type, congeneric species interactions, or both are sufficient to explain current patterns of habitat partitioning. We compare the relative importance of habitat type and congeneric interactions in each habitat and for each species to test predictions based on habitat characteristics and life-history trade-offs. Although we focus on habitat adaptation and congeneric interactions in this study, other mechanisms may also contribute to habitat partitioning in this system. For example, interactions with other species in the local community, dispersal among habitats, and/or temporal variation may influence patterns of allopatry and sympatry.

## Material and Methods

### Study system

The *M. guttatus* DC. species complex (Phrymaceae) offers a unique opportunity to examine evolutionary and ecological distributional processes because it harbors several closely related and broadly co-occurring taxa that occupy a wide range of habitat types (Vickery 1964; Wu et al. 2008). *Mimulus laciniatus* Gray is a diminutive, self-fertilizing annual that occupies seasonally drying rocky seeps in the Sierra Nevada. Throughout its range, *M. laciniatus* co-occurs with its close relative, *M. guttatus* (Vickery 1964). *Mimulus guttatus* is widely distributed throughout western North America and occupies moist habitats such as stream banks or wet meadows. Although both species can be interfertile in experimental crosses, hybridization in natural populations appears infrequent based on the high selfing rate in *M. laciniatus* and the fact that field-collected seed of both species were true-breeding in greenhouses (J. Sexton, *pers obs*.).

Our study site (Grand Bluff, 37.0771, −119.2299, 1670 m) is located where overlap between these species is common. Habitat partitioning at this site is representative of observations throughout the species' overlapping range; *M. laciniatus* and *M. guttatus* often co-occur at small spatial scales, where *M. guttatus* is often perennial and appears to occupy wetter microhabitats than *M. laciniatus* (M. Peterson, *pers. obs*.). At our study site, *M. guttatus* individuals are largest and most abundant along streams with sustained water flow throughout the year and midday shade from trees and tall grasses (Fig. 1A). *M. laciniatus* does not occur in streams, although it germinates readily in this environment (J. Sexton, *unpubl. data*). *M. laciniatus* is most abundant in moss on granite seeps inundated with snowmelt in the early spring; however, the growth season is limited because this habitat dries quickly under full sun exposure (Fig. 1B). *M. laciniatus* has deeply divided leaf margins that can ameliorate heat and drought stress in plants (Nicotra et al. 2011) and may be advantageous in fast-drying seep environments. Both species occur in lower abundance in nearby meadows (J. Sexton, *pers. obs*.) that retain high soil moisture until late summer. These three habitats differ in water and light availability, growing season length, and plant community structure (Table 1). Although not measured directly in this study, the habitats may also vary greatly in productivity. Streams appear to have the greatest biomass in both the long term (having trees and other woody plants) and within a single year (attaining largest herbaceous plant size), whereas seeps and meadows have sparser and shorter vegetation. *M. guttatus* is perennial and exhibits extensive rhizomatous growth in the stream habitat; however, it assumes an annual habit and smaller size in meadows due to seasonal drying in late summer. Despite potential differences in productivity, all habitats likely contain their own suite of environmental and biological challenges (e.g., inundation and low-light in streams, herbivory, pathogens, or soil properties) that could outweigh any fitness effects due solely to productivity differences.

**Table 1:** Site characteristics for the seep, meadow, and stream habitats. Photosynthetically active radiation (PAR) was measured (AccuPar LP-80 ceptometer; Decagon Devices, Inc.) to assess shading by neighboring vegetation in each habitat at the level of experimental individuals. Six readings were taken in each of the seep and meadow habitats, because neighboring vegetation did not reach the level of the blocks and each reading was essentially ambient light, whereas 54 readings were taken throughout the stream site to encompass variation in the herbaceous canopy. Means and standard errors (in parentheses) were calculated for each habitat. Days to soil drying after placement of experimental blocks are given for each habitat. The stream habitat remained wet throughout the year

	Seep	Meadow	Stream
PAR (μE/m^2^/sec)	1828 (52.79)	1881.5 (22.3)	909.8 (88.05)
Days to soil drying	67	95	NA

### Reciprocal transplant experiment

For each species, we pooled seed collected in 2006 from 20 individuals (>3 m apart) across the range of habitats naturally occupied at Grand Bluff. A “target-neighbor” design was used to quantify intra- and interspecific interactions. Each species was treated as a “target” in three treatments: alone, with *M. guttatus*, and with *M. laciniatus*. We planted three to four seeds (randomly thinned to one plant) from the “target” species seed pool into the center of 38 mm by 38 mm by 57 mm pots, and for neighbor treatments, we planted four to six seeds of the neighboring species around the periphery of the pot. We chose this pot size and neighbor number to simulate high competitive densities observed in field environments. Neighbors were not thinned to allow variation in neighbor density. Treatments were randomized within 66 12-cell blocks. We placed blocks in a growth chamber (14-h 23°C/10-h 4 day/night) on April 18, 2008. Upon germination, we transferred blocks to the field site (May 6, 2008). Blocks were distributed widely and set within the existing vegetation to encompass the range of conditions in each of the three habitats. We used felt squares under each block to encourage wicking, allowing plants to experience natural water availability and drying (Sexton et al. 2011). This design exposed experimental plants to habitat-specific conditions of water availability and surrounding soils as well as shading by both herbaceous and woody canopies when present (Table 1). Although cell trays may not represent a purely realistic biological environment, plants within cells attain a similar stature to adjacent un-potted plants and typically extend roots into surrounding soils like naturally growing plants. This method has been used in previous transplant studies for *M. laciniatus* (Sexton et al. 2011), and we feel that this design adequately captures major habitat differences (i.e., shading and timing of seasonal drying) while allowing for accurate tracking of individual plants and estimates of plant density. This approach also prevents damage or disturbance to the natural population of endemic *M. laciniatus* at this site as well as the moss substrate in which it grows. Semi-artificial conditions are often used within natural contexts to understand intraspecific and interspecific dynamics (e.g., Stachowicz et al. 1999) and we believe that this tray system allows a reasonable balance between creating controlled experimental conditions and providing natural growing conditions. We collected a block when all individuals were dead, or after 164 days. To evaluate overwintering performance in *M. guttatus*, we left rosettes of surviving *M. guttatus* in eight blocks from the stream habitat to overwinter.

### Fitness data

We used dry fruit mass (harvested and dried to a constant weight at 60°C for 48 h) as a fitness proxy because it is highly correlated with seed set in *M. laciniatus* (Sexton et al. 2011) and *M. guttatus* (Fenster and Ritland 1994). The actual number of neighbors varied due to differential germination, so it was analyzed as a covariate.

### Phenological data

We scored plants for phenological state every 20–25 days during flowering, and then every 3–9 weeks later in the growing season (mean 32.8 days, range 20–63 days). Individuals were scored as vegetative (no reproductive structures), flowering (including buds), fruiting (no longer flowering, but still alive), or dead (no green tissue).

### Statistical analyses

#### Fitness data

Due to the large number of individuals that failed to reproduce, we used several approaches to model differences in fitness. The first was a linear mixed model using log (dry fruit mass g + 1) as the response variable and including all individuals that failed to reproduce. To satisfy model assumptions, we fit a second linear mixed model using rank-transformed dry fruit mass (Conover and Iman 1981). Results did not differ qualitatively, so results from the rank-transformed model are presented here (referred to as total fitness model).

To distinguish early and late life-stage fitness components, survival to reproduction and fecundity were modeled separately. We used a generalized linear mixed model with a logit link function to model whether an individual survived to produce fruit (survival model). We fit a linear mixed model to examine differences in log (dry fruit mass g + 1) among reproductive individuals (fecundity model).

We fit linear mixed models using Proc Mixed, whereas generalized linear mixed models were fit using RPML in Proc Glimmix (SAS v. 9.3; SAS Institute 2011). For all models, we included species, habitat, neighbor treatment, and their two- and three-way interactions as fixed effects. We used Akaike information criteria (AIC) and likelihood ratio tests (LRT) to compare random effects structures with block and neighbor number as crossed random effects relative to models with only block. For the total fitness models, the variance due to neighbor number was estimated as zero and both AIC and LRTs supported the simpler model with only block as a random effect. Conversely, both the survivorship and the fecundity model had significant support for including number of neighbors and block as crossed random effects (LRTs for number of neighbors: χ^2^ = 10.64, df = 1, *P* = 0.0006; χ^2^ = 13.9, df = 1, *P* = 0.00019, for survivorship and fecundity models, respectively). We used the Kenward-Roger approximation for denominator degrees of freedom for all models. Given the significance of the species-by-habitat and neighbor treatment-by-habitat interactions (Table 2), we fit separate models for each habitat (Tables S1–S3). We tested all pairwise treatment differences using the Tukey-Kramer adjustment for multiple comparisons.

**Table 2:** Species, habitat, and neighbor treatment effects on fitness for three different models. Total Fitness refers to model using rank-transformed log (fruit mass g + 1); Survival refers to model using survival to reproduction as a binary variable; Fecundity refers to model using log (fruit mass g + 1) only for those individuals that reproduced. *P*-values less than 0.05 are in bold. See Material and Methods for statistical details

	Total Fitness				Survival				Fecundity			
			
Source	Df1	Df2	F	Pr(≥|F|)	Df1	Df2	F	Pr(≥|F|)	Df1	Df2	F	Pr(≥|F|)
Habitat	2	60	9.12	**0.0003**	2	84.06	7.75	**0.0008**	2	72.4	3.74	**0.0284**
Species	1	508	17.41	**<0.0001**	1	562	31.01	**<0.0001**	1	218	0.40	0.5285
Treatment	2	508	20.63	**<0.0001**	2	562	16.88	**<0.0001**	2	214	0.04	0.9635
Habitat* Species	2	508	7.05	**0.001**	2	562	4.10	**0.0171**	2	226	4.64	**0.0107**
Habitat* Treatment	4	508	2.88	**0.0221**	4	562	2.42	**0.0477**	4	220	1.46	0.2157
Species* Treatment	2	509	0.13	0.8821	2	562	1.10	0.3334	2	217	1.31	0.2717
Habitat* Species* Treatment	4	509	0.57	0.6822	4	562	0.37	0.8274	4	215	0.41	0.8022

Because the effects of species interactions are captured by the differences between neighbor and alone treatments, rather than the neighbor treatment itself, we devised *t*-tests for ten *a priori* contrasts (Proc Glimmix in SAS) related to species interactions. Each neighbor treatment was contrasted with the alone treatment, regardless of habitat and target species, to test for a general effect of intraspecific and interspecific interactions (Table S4, contrasts 1–2); this effect was then contrasted across species (contrasts 3–4) and habitats (contrasts 5–10) (see Table S4 for statistical details).

#### Phenological data

We examined differences in time to flowering using Cox Proportional Hazards models to allow inclusion of censored individuals that died before flowering could be observed. Given the extreme differences in the timing and magnitude of censoring among habitats (due to differences in the timing of seasonal drying; see Table 1), we fit separate models (species, neighbor treatment, and their interaction) for each habitat. We fit models with block and neighbor number as crossed random effects, block only, or no random effects. Results did not differ qualitatively among models, so we used models without random effects to allow multiple comparison adjustments for testing treatment differences. Significance of fixed effects was tested using likelihood ratio tests. Models were fit using the Coxme package (Therneau 2011) in R 2.9.2 (R Core Development Team 2011). Tukey-adjusted multiple comparisons used the Multcomp package (Hothorn et al. 2008) in R.

## Results

### Fitness

**Figure 2:**
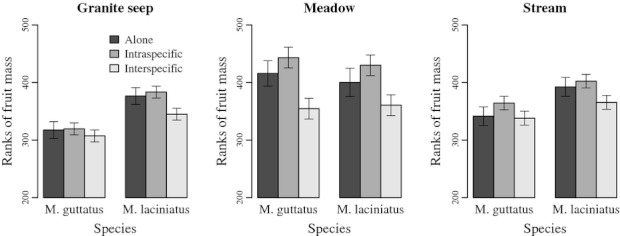
Least square means ± se for rank of total fitness, where total fitness is log (fruit mass g + 1) for all experimental individuals.

**Figure 3:**
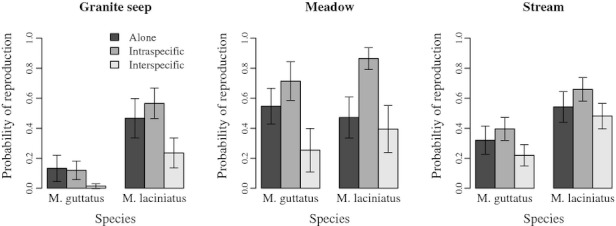
Least square means ± se for probability of survival to reproduction.

**Figure 4:**
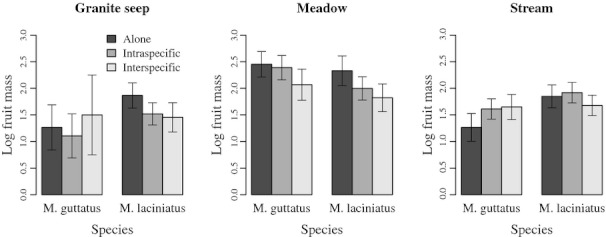
Least square means ± se for fecundity, calculated as log (fruit mass g + 1), only for those individuals that produced fruit.

Species interactions (i.e., neighbor treatment) and habitat had significant effects on total fitness for both *M. guttatus* and *M. laciniatus* (Fig. 2, Table 2). However, species interactions primarily affected survival, whereas habitat had significant effects on both survival and fecundity (Figs. 3, 4, Table 2).

Survival in *M. guttatus* was highly dependent on habitat (*F*_2,304.2_ = 8.56, *P* = 0.0002). *M. guttatus* individuals in the seep were 3.58 times less likely to survive to reproduction than individuals in the meadow habitat, and 2.98 times less likely than individuals in the stream habitat [Fig. 3; 95% CI for odds ratios: seep/meadow (1.84, 6.99), seep/stream (1.59, 5.59)]. There was no significant difference in survival between the meadow and stream habitats for either species (*M. guttatus: t*_167.9_ = 1.44, *P* = 0.7012; *M. laciniatus*: *t*_213.2_ = −0.29, *P* = 0.9997). The probability of survival for *M. laciniatus* did not differ among habitats (*F*_2,183.8_=1.05, *P* = 0.3524). Fecundity followed the same general pattern, with strong habitat effects for *M. guttatus,* but not *M. laciniatus* (Fig. 4; *M*. *guttatus*: *F*_2,127_ = 6.57, *P* = 0.0019; *M*. *laciniatus*: *F*_2,75.5_ = 0.99, *P* = .3782). *M. guttatus* individuals produced significantly more seed in the meadow than in the stream habitat (*t*_83.3_ = 3.48, *P* = 0.0078).

Overall, interspecific interactions were competitive (total fitness: *t*_507.5_ = 3.28, *P* = 0.0011; survival: *t*_562_ = 2.99, *P* = 0.0029; fecundity: *t*_221.5_ = 0.26, *P* = 0.7930), whereas intraspecific interactions were marginally facilitative (total fitness: *t*_508_ =1.88, *P* = 0.0607; survival: *t*_562_ =1.91, *P* = 0.0572; fecundity: *t*_228.3_ =0.23, *P* = 0.8208) (Fig. 4). The effect of species interactions differed among habitats (Table 2). Interspecific competition was more intense in the meadow habitat relative to the stream habitat for fecundity (*t*_216.1_ =2.08, *P* = 0.0388), and marginally so for survival (*t*_562_ =1.68, *P* = 0.0936) and total fitness (*t*_507.9_ =1.71, *P* = 0.0872) (Fig. 4). However, the intensity of intraspecific interactions did not differ statistically among habitats for any fitness components (Tables S1–S3). There was no evidence that *M. guttatus* and *M. laciniatus* differed in the intensity of either interspecific (total fitness: *t*_509.2_ = −0.43, *P* = 0.6659; survival: *t*_562_ = 1.41, *P* = 0.1587; fecundity: *t*_215.2_ = −1.58, *P* = 0.1149) or intraspecific interactions (total fitness: *t*_509.7_ = −0.10, *P* = 0.9176; survival: *t*_562_ = 1.18, *P* = .2397; fecundity: *t*_215.2_ = −1.17, *P* = 0.2438).

In the seep, there were strong differences in survival between *M. guttatus* and *M. laciniatus* (*F*_1, 144_ = 19.54, *P* < 0.0001). *M. laciniatus* was 10.53 times more likely to survive to reproduction than *M. guttatus* (95% CI for odds ratio: 3.68, 30.30). Interspecific competition was strong as individuals grown alone were 5.47 times more likely to survive to reproduction than those in interspecific treatments (95% CI for odds ratio: 1.04, 28.81); there was no significant difference between alone and intraspecific treatments. The strength of interspecific competition did not differ between *M. guttatus* and *M. laciniatus* (*F*_2,144_ = 0.42, *P* = 0.6574). There were no significant effects of species identity (*F*_1,28.2_ = 1.12, *P* = 0.2993) or neighbor treatment (*F*_2,27.1_ = 0.34, *P* = 0.7149) on fecundity.

In the meadow habitat, the relative performance of either species was dependent on the dominant neighboring species. *M.* *laciniatus* with intraspecific neighbors outperformed *M. guttatus* with interspecific neighbors in both total fitness and survival (total fitness: *t*_157_ = 3.79, *P* = 0.0029; survival: *t*_174_ = 4.3, *P* = 0.0004) (Figs. 4). Similarly, *M. guttatus* with intraspecific neighbors outperformed *M. laciniatus* with interspecific neighbors in total fitness (*t*_157_ = 4.14, *P* = 0.0008), with a marginally significant difference in survival (*t*_174_ = 2.61, *P* = 0.10). There were no species differences in either total fitness (*t*_158_ = .57, *P* = 0.5712) or survival (*t*_174_ = 1.18, *P* = 0.2398), although *M. guttatus* did exhibit marginally greater fecundity (*t*_79.6_ = 1.84, *P* = 0.0696). Rather, species interactions had strong effects on total fitness (*F*_2,157_ = 15.95, *P* < 0.0001) and survival (*F*_2,16.92_ = 12.96, *P* = 0.0004). Individuals in intraspecific treatments were 3.86 times more likely to survive to reproduction than individuals grown alone, and 8.47 times more than individuals grown with interspecific competitors [95% CI for odds ratios: intraspecific/alone (1.06, 13.89), intraspecific/interspecific (3.58, 20)]. There was no evidence that the strength of these interactions differed between species for either total fitness (*F*_2,158_ = 0.30, *P* = 0.7412) or survival (*F*_2,174_ = 0.94, *P* = 0.3921).

In the stream habitat, *M. laciniatus* outperformed *M. guttatus* in total fitness (*F*_1, 220_ = 14.25, *P* = 0.0002), survival (*F*_1,244_ = 14.03, *P* = 0.0002), and fecundity (*F*_1,102_ = 5.46, *P* = 0.0214). *M. laciniatus* was 2.9 times more likely to survive to reproduction than *M. guttatus* (95% CI for odds ratio: 1.66, 5.08), and had greater first year fecundity (*t*_102_ = 2.34, *P* = 0.0214). However, the subset of *M. guttatus* individuals left to overwinter in this habitat grew larger in the second year than *M. laciniatus* grows in any of the local habitats (J. Sexton, *unpubl. data*). Additionally, species interactions affected the probability of survival (*F*_2,244_=3.29, *P* = 0.0388), but not fecundity (*F*_2,104_=.73, *P* = 0.4843). Individuals in intraspecific treatments were 2.2 times more likely to survive to reproduction than individuals in interspecific treatments (95% CI for odds ratio: 1.19, 4.07). The strength of this effect did not differ between species (*F*_2,244_ = 0.07, *P* = 0.9354).

#### Phenology

**Figure 5:**
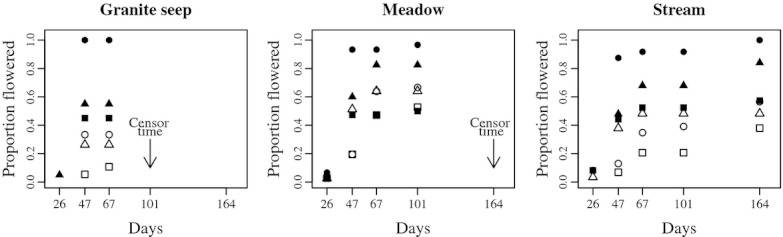
Cumulative proportion of individuals that have flowered through time for each site, species, and neighbor treatment. Arrows indicate date at which individuals were removed from site due to seasonal drying, causing individuals that had not yet flowered to be right-censored. Filled symbols are *Mimulus laciniatus*, open symbols are *Mimulus guttatus*. Neighbor treatments are as follows: ▪ = alone; • = intraspecific; ▲= interspecific.

There were significant differences in flowering time among species, with *M. laciniatus* having a higher probability of flowering in all three habitats (Fig. 5; Seep: χ^2^ = 25.354, *P* < 0.0001; Meadow: χ^2^ = 17.353, *P* < 0.0001; Stream: χ^2^ = 30.831, *P* < 0.0001). Neighbor effects on flowering were significant in all three habitats; the presence of either inter- or intraspecific neighbors increased the probability of flowering relative to treatments without neighbors (Fig. 5; Seep: χ^2^ = 13.087, *P* = 0.0014; Meadow: χ^2^ = 17.998, *P* = 0.0001; Stream: χ^2^ = 20.933, *P* < 0.0001). In the meadow habitat, there was a significant species-by-neighbor treatment interaction, with *M. laciniatus* flowering earlier in response to neighbors than *M. guttatus* (χ^2^ = 10.85, *P* = 0.0044).

## Discussion

### Determinants of niche boundaries between close relatives

We examined habitat partitioning and intra- and interspecific interactions between two close relatives within a species complex to understand factors setting local limits and allowing coexistence. Specifically, we tested whether habitat performance, congeneric interactions, or both were sufficient to explain current species distributions. Alternatively, interactions with more distantly related species in the community and/or fluctuation-dependent coexistence mechanisms may contribute to observed niche boundaries.

Habitat partitioning is clear in these recently diverged species, with strong lifetime fitness costs for *M. guttatus* in the seep habitat dominated by *M.* *laciniatus*. Low absolute fitness of *M. guttatus*, even in the absence of competition, suggests that severe drought stress is the primary factor limiting establishment of *M. guttatus* in seeps. However, species interactions reinforce this barrier, as immigrating *M. guttatus* are faced with severe interspecific competition. These results corroborate our field observations that *M. guttatus* does not invade seeps occupied by *M.* *laciniatus*, despite the close proximity (<5 m) between stream and seep habitats.

In contrast, neither species is dominant, either intrinsically or competitively, in the sympatric meadow habitat. Rather, either species becomes competitively dominant when abundant, through a combination of intraspecific facilitation and suppression of interspecific competitors. In the absence of temporal variation or dispersal among habitats, these interactions suggest competitive exclusion of whichever species is initially less abundant. Given that fluctuation-independent interactions did not explain the observed field pattern of species coexistence, fluctuation-dependent mechanisms (e.g., the storage effect) may explain coexistence of these close relatives in the meadow habitat.

Interestingly, *M. laciniatus* seedlings outperformed *M. guttatus* seedlings in the stream habitat in both survival and fecundity; this is in contradiction with the observed absence of *M. laciniatus* in this habitat*. M. laciniatus* is not limited from establishing in the stream habitat by either seed germination (J. Sexton, *unpubl. data*) or competition with *M. guttatus* seedlings (this study). Given the relatively tall, dense vegetation in stream habitats, we suggest that *M. laciniatus* may instead be limited by interactions with neighboring plant species. Although seedlings in this experiment were grown in blocks set within the much taller herbaceous canopy, seedlings did not directly compete with surrounding vegetation for space. *M. laciniatus* individuals naturally occurring in this habitat would likely experience strong asymmetric competition from other plant species, including mature *M. guttatus*. Furthermore, observations of *M. guttatus* at this site confirm that perennial individuals grow and reproduce over several years, including those individuals left to overwinter. Iteroparous, herbaceous perennial species generally exhibit reduced seedling survival relative to annuals (e.g., Silvertown et al. 1993), which may explain why measuring seedling performance alone failed to predict the dominance of perennial *M. guttatus* in stream habitats. Given the relatively low seedling survival of *M. guttatus* in all three habitats, streams may act as a refuge for *M. guttatus* recruits by fostering high adult survival. We expect that *M. laciniatus* is probably excluded from streams by surrounding vegetation, but we acknowledge that this remains to be verified experimentally.

### Habitat heterogeneity and species coexistence

We tested whether intra- and interspecific interactions explained the coexistence of two close relatives in the sympatric meadow habitat. We did not find evidence of fluctuation-independent mechanisms for species coexistence (Chesson 2000); intraspecific competition was not stronger than interspecific competition for either species. However, fluctuation-independent mechanisms may still operate if intraspecific facilitation and interspecific competition results in spatial clustering of each species. While we did not observe clear species clustering among mature plants in the meadow habitat, this mechanism could theoretically promote coexistence at earlier life stages (e.g., germination and/or among seedlings).

In the absence of clear fluctuation-independent mechanisms, this study supports the growing consensus that habitat heterogeneity can promote species coexistence via fluctuation-dependent mechanisms (Chesson 2000; Amarasekare 2003). Our results support the potential for a spatial storage effect, as both species are effectively released from competition in each specialized habitat that, in turn, acts as a source for dispersal into other habitat types. Continual propagule pressure from the seep habitat for *M. laciniatus* and from the stream habitat for *M. guttatus*, which host relatively large numbers of each species, may act to prevent competitive exclusion of either species in the meadow habitat. Among-habitat dispersal is likely because seed dispersal in *Mimulus* occurs easily over hundreds of meters (Vickery 1999). Although single-year monitoring did not adequately capture lifetime fitness for *M. guttatus* in the stream habitat, field observations of the abundance and large size of mature *M. guttatus* support the argument that the perennial habit adopted in streams establishes a demographic stronghold for this species.

Additionally, inter-annual variation in the strength of species interactions (e.g., mediated though inter-annual differences in water availability) may result in a temporal storage effect that contributes to species coexistence in the meadow habitat (Chesson and Warner 1981). However, in order for temporal environmental variation to result in stable coexistence for annual species, each species must exhibit a persistent seed bank and species-specific germination responses to environmental conditions (Pake and Venable 1996; Angert et al. 2009). There is little evidence that such conditions are met for either *M. guttatus* or *M. laciniatus*; both species exhibit high germination rates in moist conditions (J. Sexton, *pers. obs*.) and *M. guttatus* is known to establish only transient (2–3 years), low-quantity seed banks (Vickery 1999; Truscott et al. 2006). For this reason, it is unlikely that temporal variation alone maintains long-term species coexistence in the meadow habitat; however, it is likely a contributing mechanism for short-term coexistence.

### Intensity versus importance of interactions

The seep, meadow, and stream habitats differ in the severity of drought stress, the length of the growth season, and the abundance and size of neighboring vegetation. Together, these habitats may represent a gradient of community productivity. However, all habitats present unique and unknown stressors (e.g., pathogens, herbivory, scouring, etc.) regardless of productivity differences. If these habitats create a productivity gradient, we found little support for the hypothesis that the intensity of competition between congeners increases with productivity. The absolute strength of interspecific competition between congeners was greater among plants in the meadow than in the stream habitat where plants had the longest period for growth. Furthermore, the direction of species interactions remained constant across habitats. Interspecific interactions were always competitive, whereas intraspecific interactions were always facilitative.

If one assumes that the three habitats represent a productivity gradient, a consideration of the relative importance of species interactions (*sensu* Welden and Slauson 1986) supports competitive, but not facilitative, predictions of the SGH. In the seep habitat, species interactions were relatively unimportant given the strong effects of seasonal drying. The low absolute fitness of *M. guttatus* in seeps rendered any effect of species interactions relatively unimportant in determining species performance, even though the absolute intensity of these interactions were comparable to other habitats. Conversely, species interactions were important in the meadow habitat, where intrinsic species differences were lacking and relative performance was determined by the balance of intraspecific facilitation and interspecific competition. Species interactions between *M. laciniatus* and *M. guttatus* seedlings were of intermediate importance in the stream habitat, because the intrinsic advantage of *M. laciniatus* over *M. guttatus* seedlings at this site was ameliorated when *M. guttatus* seedlings experienced intraspecific facilitation and acted as a competitor to *M. laciniatus*. However, habitat performance and interactions with *M. guttatus* seedlings do not explain the persistent exclusion of *M. laciniatus* from the stream habitat, suggesting that competitive interactions with mature *M. guttatus* or other neighboring vegetation may maintain this niche boundary. Conversely, coexistence of both species in the meadow habitat suggests that species interactions, while intense, are insufficient to cause competitive exclusion of either species in the face of immigration from other habitat types.

Quantitative tests for the importance of competition for population-level outcomes, such as the likelihood or speed of competitive exclusion (Chesson and Huntly 1997; Violle et al. 2010), require long-term data (Freckleton et al. 2009). However, our results are consistent with predictions that the intensity of species interactions may be uncorrelated with importance across environmental gradients (Welden and Slauson 1986; Gaucherand et al. 2006) and emphasize the value of testing species interactions across a range of field environments. The direction of species interactions can vary between field and greenhouse environments (e.g., Burns and Strauss 2011) and life-history stages (e.g., Leger and Espeland 2010). Here, the relative importance of species interactions changed among local habitats. Consideration of both the intensity and importance of species interactions should provide insight to the long-standing debate on whether competition is stronger in more productive or stressful environments (Grime 1977; Tilman 1987; Grace 1991; Violle et al. 2010).

### Niche evolution between close relatives

As predicted, we found evidence for habitat partitioning between these closely related species. We detected critical fitness differences between species in response to drought stress in the allopatric seep habitat, but did not detect any differences in performance within the sympatric meadow habitat. Additionally, interspecific competition was most intense in the meadow habitat, further suggesting that species differences are minimized in this habitat of niche overlap.

Surprisingly, we found little evidence for a trade-off in habitat performance in the more specialized, geographically restricted species (*M. laciniatus*); individuals exhibited similar performance across all three habitats. Although we did not detect differences in competitive ability between congeners, several other lines of evidence suggest that niche divergence between these closely related species may have been driven by a stress tolerance – competitive ability trade-off. *M. guttatus*, which exhibits several classically competitive traits (e.g., large size due to rhizomatous growth and perennial life-history) (Keddy et al. 2002), was unable to tolerate drought stress associated with the seep habitat. The evolution of an obligate annual life history as a drought avoidance strategy in *M. laciniatus* may have come at the cost of reduced competitive ability in the thickly vegetated stream habitat, potentially explaining this persistent niche boundary. The geographically restricted *M.* *laciniatus* appears to have a wider habitat tolerance than *M. guttatus*, at least in the absence of community-wide competition; this suggests that the mechanism restricting range size in *M. laciniatus* involves community-level effects, not necessarily abiotic conditions such as water availability. Our results support the hypothesis that the colonization of rare, stressful seep habitats by *M. laciniatus* has led to an annual life form with reduced capacity to compete with larger perennials occupying more widely distributed stream environments.

### Summary

Although limited conclusions can be drawn from a single system, these recently diverged *Mimulus* species provide insight into the process of niche divergence and the role of competition across a range of environmental quality. Niche partitioning among habitats is driven by physiological tolerance limits in *M. guttatus*, and likely driven by community-level competitive limitations in *M. laciniatus*, suggesting trade-offs between adaptations to local environments. Despite strong habitat-specific signals of niche partitioning, we found no fitness differences in the sympatric meadow habitat. This pattern of fitness and competitive equivalency in the meadow habitat suggest that coexistence here is maintained by fluctuation-dependent mechanisms, such as the storage effect. Our examination of these factors among locally sympatric and allopatric habitats offered insights into how species distributions can be shaped by deterministic adaptive processes in specialized environments and by stochastic processes in shared environments. More experimental work is needed to test these generalities across a variety of recently diverged taxa.

## Supporting Information

**Table S1.** Results from linear mixed models for total fitness fit for each habitat separately.

**Table S2.** Results from GLMMs for survival to reproduction fit for each habitat separately.

**Table S3.** Results from linear mixed models for fecundity fit for each habitat separately.

**Table S4.** Results of *a priori* contrasts of LS means.

## References

[b1] Amarasekare P (2003). Competitive coexistence in spatially structured environments: a synthesis. Ecol. Lett.

[b2] Angert AL, Huxman TE, Chesson P, Venable DL (2009). Functional tradeoffs determine species coexistence via the storage effect. Proc. Natl Acad. Sci.

[b3] Bertness MD, Callaway R (1994). Positive interactions in communities. Trends Ecol. Evol.

[b4] Burns JH, Strauss SY (2011). More closely related species are more ecologically similar in an experimental test. Proc. Natl Acad. Sci. USA.

[b5] Callaway RM (1995). Positive interactions among plants. Bot. Rev.

[b6] Chesson P (2000). Mechanisms of maintenance of species diversity. Annu. Rev. Ecol. Syst.

[b7] Chesson P, Huntly N (1997). The roles of harsh and fluctuating conditions in the dynamics of ecological communities. Am. Nat.

[b8] Chesson PL, Warner RR (1981). Environmental variability promotes coexistence in lottery competitive systems. Am. Nat.

[b9] Choler P, Michalet R, Callaway RM (2001). Facilitation and competition on gradients in alpine plant communities. Ecology.

[b10] Connell JH (1961). The influence of interspecific competition and other factors on the distribution of the barnacle chthamalus stellatus. Ecology.

[b11] Conover WJ, Iman RL (1981). Rank transformations as a bridge between parametric and nonparametric statistics. Am. Stat.

[b12] Cui B, He Q, Zhang K, Chen X (2011). Determinants of annual–perennial plant zonation across a salt–fresh marsh interface: a multistage assessment. Oecologia.

[b13] Emery NC, Ewanchuk PJ, Bertness MD (2001). Competition and salt-marsh plant zonation: stress tolerators may be dominant competitors. Ecology.

[b14] Ettinger AK, Ford KR, HilleRisLambers J (2011). Climate determines upper, but not lower, altitudinal range limits of Pacific Northwest conifers. Ecology.

[b15] Evans Margaret EK, Smith Stephen A, Flynn Rachel S, Donoghue Michael J (2009). Climate, niche evolution, and diversification of the “Bird-Cage” evening primroses (Oenothera, Sections Anogra and Kleinia). Am. Nat.

[b16] Fenster CB, Ritland K (1994). Evidence for natural selection on mating system in Mimulus (Scrophulariaceae). Int. J. Plant Sci.

[b17] Freckleton RP, Watkinson AR, Rees M (2009). Measuring the importance of competition in plant communities. J. Ecol.

[b18] Gaucherand S, Liancourt P, Lavorel S (2006). Importance and intensity of competition along a fertility gradient and across species. J. Veg. Sci.

[b19] Goldberg DE (1996). Competitive ability: definitions, contingency and correlated traits. Phil. Trans. Biol. Sci.

[b20] Grace JB (1991). A clarification of the debate between Grime and Tilman. Funct. Ecol.

[b21] Grime JP (1977). Evidence for the existence of three primary strategies in plants and its relevance to ecological and evolutionary theory. Am. Nat.

[b22] Gross SJ, Price TD (2000). Determinants of the Northern and Southern Range limits of a warbler. J. Biogeogr.

[b23] Holt RD (2009). Bringing the Hutchinsonian niche into the 21st century: ecological and evolutionary perspectives. Proc. Natl Acad. Sci. USA.

[b24] Hothorn T, Bretz F, Westfall P (2008). Simultaneous inference in general parametric models. Biom. J.

[b25] Hutchinson GE (1957). Population studies – animal ecology and demography – concluding remarks. Cold Spring Harb. Symp. Quant. Biol.

[b26] Keddy P, Nielsen K, Weiher E, Lawson R (2002). Relative competitive performance of 63 species of terrestrial herbaceous plants. J. Veg. Sci.

[b27] Kirkpatrick M, Barton NH (1997). Evolution of a species' range. Am. Nat.

[b28] Leger EA, Espeland EK (2010). The shifting balance of facilitation and competition affects the outcome of intra- and interspecific interactions over the life history of California grassland annuals. Plant Ecol.

[b29] Liancourt P, Tielbörger K (2009). Competition and a short growing season lead to ecotypic differentiation at the two extremes of the ecological range. Funct. Ecol.

[b30] Liancourt P, Callaway RM, Michalet R (2005). Stress tolerance and competitive-response ability determine the outcome of biotic interactions. Ecology.

[b31] MacArthur R, Levins R (1967). Limiting similarity convergence and divergence of coexisting species. Am. Nat.

[b32] Maestre FT, Callaway RM, Valladares F, Lortie CJ (2009). Refining the stress-gradient hypothesis for competition and facilitation in plant communities. J. Ecol.

[b33] Nicotra AB, Leigh A, Boyce CK, Jones CS, Niklas KJ, Royer DL (2011). The evolution and functional significance of leaf shape in the angiosperms. Funct. Plant Biol.

[b34] Pake CE, Venable DL (1996). Seed banks in desert annuals: implications for persistence and coexistence in variable environments. Ecology.

[b35] R Core Development Team (2011). R: a language and environment for statistical computing.

[b36] Rundle HD, Nosil P (2005). Ecological speciation. Ecol. Lett.

[b37] SAS Institute (2011). SAS for Windows version 9.3.

[b38] Sears ALW, Chesson P (2007). New methods for quantifying the spatial storage effect: an illustration with desert annuals. Ecology.

[b39] Sexton JP, McIntyre PJ, Angert AL, Rice KJ (2009). Evolution and ecology of species range limits.

[b40] Sexton JP, Strauss SY, Rice KJ (2011). Gene flow increases fitness at the warm edge of a species' range. Proc. Natl Acad. Sci. USA.

[b41] Silvertown J, Franco M, Pisanty I, Mendoza A (1993). Comparative plant demography–relative importance of life-cycle components to the finite rate of increase in woody and herbaceous perennials. J. Ecol.

[b42] Stachowicz JJ, Whitlatch RB, Osman RW (1999). Species diversity and invasion resistance in a marine ecosystem. Science.

[b43] Tercek MT, Whitbeck JL (2004). Heat avoidance life history strategy controls the distribution of geothermal agrostis in yellowstone. Ecology.

[b44] Therneau T (2011). coxme: Mixed Effects Cox Models. R package version 2.1-3.

[b45] Tilman D (1982). Resource Competition and Community Structure.

[b46] Tilman D (1987). On the meaning of competition and the mechanisms of competitive superiority. Funct. Ecol.

[b47] Tilman D (2004). Niche tradeoffs, neutrality, and community structure: a stochastic theory of resource competition, invasion, and community assembly. Proc. Natl Acad. Sci. USA.

[b48] Truscott AM, Soulsby C, Palmer SCF, Newell L, Hulme PE (2006). The dispersal characteristics of the invasive plant Mimulus guttatus and the ecological significance of increased occurrence of high-flow events. J. Ecol.

[b49] Vickery RK (1964). Barriers to gene exchange between members of the Mimulus guttatus complex (Scrophulariaceae). Evolution.

[b50] Vickery RK (1999). Remarkable waxing, waning, and wandering of populations of *Mimulus guttatus*: an unexpected example of global warming. Great Basin Nat.

[b51] Violle C, Pu Z, Jiang L (2010). Experimental demonstration of the importance of competition under disturbance. Proc. Natl Acad. Sci.

[b52] Welden CW, Slauson WL (1986). The intensity of competition versus its importance: an overlooked distinction and some implications. Q. Rev. Biol.

[b53] Wethey DS (1983). Geographic limits and local zonation: the barnacles *Semibalanus**Balanus*) and *Chthamalus* in New England. Biol. Bull.

[b54] Wu CA, Lowry DB, Cooley AM, Wright KM, Lee YW, Willis JH (2008). Mimulus is an emerging model system for the integration of ecological and genomic studies. Heredity.

