# Supplementary material for: Niche partitioning between close relatives suggests trade-offs between adaptation to local environments and competition
Source: Ecol Evol. 2013 Jan 24;3(3):512–22. doi: 10.1002/ece3.462 (PMC3605842; doi:10.1002/ece3.462)
Supplement: Supplementary file 1 [file ece30003-0512-SD1.doc]

Table S1: ANOVA tables for linear mixed models examining rank-transformed total fitness for each habitat

**Seep** **Meadow**  **Stream**

**Source Df1 Df2 F Pr(>|F|) Df1 Df2 F Pr(>|F|) Df1 Df2 F Pr(>|F|)**

Species 1 144 30.02 **<.0001** 1 158 0.32 0.5712 1 220 14.25 **0.0002**

Treatment 2 144 3.23 **0.0424** 2 157 15.95 **<.0001** 2 221 4.24 **0.0156**

Species * Treatment 2 144 0.85 0.4297 2 158 0.30 0.7412 2 221 0.40 0.6730

Table S1: Species and neighbor treatment effects on rank of total fitness (log[fruit mass g +1] for all experimental individuals, see methods for details). Separate models were fit for each habitat. P-values less than 0.1 are in bold.
