# Supplementary material for: Niche partitioning between close relatives suggests trade-offs between adaptation to local environments and competition
Source: Ecol Evol. 2013 Jan 24;3(3):512–22. doi: 10.1002/ece3.462 (PMC3605842; doi:10.1002/ece3.462)
Supplement: Supplementary file 2 [file ece30003-0512-SD2.doc]

Table S2: ANOVA tables for generalized linear mixed models examining probability of survival to reproduction for each habitat

**Seep** **Meadow**  **Stream**

**Source Df1 Df2 F Pr(>|F|) Df1 Df2 F Pr(>|F|) Df1 Df2 F Pr(>|F|)**

Species 1 144 19.54 **<.0001** 1 174 1.39 0.2398 1 244 14.03 **0.0002**

Treatment 2 144 3.86 **0.0233** 2 16.92 12.96 **0.0004** 2 244 3.29 **0.0388**

Species * Treatment 2 144 0.42 0.6574 2 174 0.94 0.3921 2 244 0.07 0.9354

Table S2: Species and neighbor treatment effects on survival (probability of survival to reproduction, see methods for details). Separate models were fit for each habitat. P-values less than 0.1 are in bold.
