# Supplementary material for: Niche partitioning between close relatives suggests trade-offs between adaptation to local environments and competition
Source: Ecol Evol. 2013 Jan 24;3(3):512–22. doi: 10.1002/ece3.462 (PMC3605842; doi:10.1002/ece3.462)
Supplement: Supplementary file 3 [file ece30003-0512-SD3.doc]

Table S3: ANOVA tables for linear mixed models examining fecundity for each habitat

**Seep** **Meadow**  **Stream**

**Source Df1 Df2 F Pr(>|F|) Df1 Df2 F Pr(>|F|) Df1 Df2 F Pr(>|F|)**

Species 1 28.2 1.12 0.2993 1 79.6 3.38 **0.0696** 1 102 5.46 **0.0214**

Treatment 2 27.1 0.34 0.7149 2 79.8 1.88 0.1597 2 104 0.73 0.4843

Species * Treatment 2 30.6 0.33 0.7199 2 78 0.38 0.6838 2 102 1.26 0.2870

Table S3: Species and neighbor treatment effects on fecundity (log[fruit mass g +1] for individuals that reproduced, see methods for details). Separate models were fit for each habitat. P-values less than 0.1 are in bold.
