# Supplementary material for: Niche partitioning between close relatives suggests trade-offs between adaptation to local environments and competition
Source: Ecol Evol. 2013 Jan 24;3(3):512–22. doi: 10.1002/ece3.462 (PMC3605842; doi:10.1002/ece3.462)
Supplement: Supplementary file 4 [file ece30003-0512-SD4.doc]

Table S4: *A priori* contrasts of LS means for each of three models

**Total Fitness** **Survival**  **Fecundity**

**Contrast Est. DF t Pr(>|t|) Est. DF t Pr(>|t|) Est. DF t Pr(>|t|)**

**Intraspecific** 16.49 508 1.88 **.0607** 0.55 562 1.91 **.0572** -0.03 228.3 -0.23 .8208

**Interspecific** 28.86 507.5 3.28 **.0011** 1.05 562 2.99 **.0029** 0.05 221.5 0.26 .7930

**Species * Intraspecific** -1.82 509.7 -0.10 .9176 0.64 562 1.18 .2397 -0.29 215.2 -1.17 .2438

**Species * Interspecific** -7.61 509.2 -0.43 .6659 0.86 562 1.41 .1587 -0.52 215.2 -1.58 .1149

**Habitat * Intraspecific:**

Meadow vs. Seep 24.22 507.9 1.06 .2900 0.65 562 0.90 .3661 -0.08 226.5 -0.22 .8290

Meadow vs. Stream 12.57 508.6 0.62 .5359 0.53 562 0.90 .3659 -0.40 209.6 -1.75 **.0819**

Seep vs. Stream -11.65 507.5 -0.55 .5833 -0.12 562 -0.18 .8606 -0.33 227.7 -0.94 .3463

**Habitat * Interspecific:**

Meadow vs. Seep 29.54 507.5 1.29 .1973 -0.06 562 -0.07 .9471 0.61 219.3 1.31 .1903

Meadow vs. Stream 34.90 507.9 1.71 **.0872** 0.96 562 1.68 **.0936** 0.55 216.1 2.08 **.0388**

Seep vs. Stream 5.36 507.3 0.25 .8013 1.01 562 1.26 .2084 -0.07 220.5 -0.14 .8859

Table S4: Results of *a priori* contrasts of LS means. Contrasts were tested for each of three models (see methods for statistical details). Est. is the estimate of the difference between LS means. P-values less than 0.1 are in bold. **Intraspecific**: the fitness of plants grown alone was contrasted with plants grown with conspecific neighbors regardless of habitat and target species. **Interspecific**: the fitness of plants grown alone was contrasted with plants grown with heterospecific neighbors regardless of habitat and target species. **Species * Intraspecific**: the difference in fitness of individuals grown alone relative to individuals grown with conspecifics was contrasted for *M. guttatus* vs. *M. laciniatus* regardless of habitat. **Species * Interspecific**: the difference in fitness of individuals grown with heterospecifics relative to individuals grown alone was contrasted for *M. guttatus* vs. *M. laciniatus* regardless of habitat. **Habitat * Intraspecific**: the difference in fitness of individuals grown alone relative to individuals grown with conspecifics was contrasted for each pair of habitats regardless of target species. **Habitat * Interspecific**: the difference in fitness of individuals grown with heterospecifics relative to individuals grown alone was contrasted for each pair of habitats regardless of target species.
